# Management of a caseous lymphadenitis outbreak in a new Iberian ibex (*Capra pyrenaica*) stock reservoir

**DOI:** 10.1186/s13028-014-0083-x

**Published:** 2014-12-10

**Authors:** Andreu Colom-Cadena, Roser Velarde, Jesús Salinas, Carmen Borge, Ignacio García-Bocanegra, Emmanuel Serrano, Diana Gassó, Ester Bach, Encarna Casas-Díaz, Jorge R López-Olvera, Santiago Lavín, Luís León-Vizcaíno, Gregorio Mentaberre

**Affiliations:** Servei d’Ecopatologia de Fauna Salvatge (SEFaS), Departament de Medicina i Cirurgia Animals, Facultat de Veterinària, Universitat Autònoma de Barcelona (UAB), 08193 Bellaterra, Spain; Departamento de Sanidad Animal, Facultad de Veterinaria, Universidad de Murcia (Regional Campus of International Excellence “Campus Mare Nostrum”), Murcia, Spain; Departamento de Sanidad Animal, Facultad de Veterinaria, Universidad de Córdoba - Agrifood Excellence International Campus (ceiA3), Córdoba, Spain; CESAM, Departamento de Biologia, Universidade de Aveiro, 3810-193 Aveiro, Portugal

**Keywords:** *Capra pyrenaica*, *Corynebacterium pseudotuberculosis*, Caseous lymphadenitis, Antibodies, Autovaccine, Acute phase proteins

## Abstract

**Background:**

In 2010, an Iberian ibex (*Capra pyrenaica hispanica*) stock reservoir was established for conservation purposes in north-eastern Spain. Eighteen ibexes were captured in the wild and housed in a 17 hectare enclosure. Once in captivity, a caseous lymphadenitis (CLA) outbreak occurred and ibex handlings were carried out at six-month intervals between 2010 and 2013 to perform health examinations and sampling. Treatment with a bacterin-based autovaccine and penicillin G benzatine was added during the third and subsequent handlings, when infection by *Corynebacterium pseudotuberculosis* was confirmed. Changes in lesion score, serum anti-*C. pseudotuberculosis* antibodies and haematological parameters were analyzed to assess captivity effects, disease emergence and treatment efficacy. Serum acute phase proteins (APP) Haptoglobin (Hp), Amyloid A (SAA) and Acid Soluble Glycoprotein (ASG) concentrations were also determined to evaluate their usefulness as indicators of clinical status.

Once in captivity, 12 out of 14 ibexes (85.7%) seroconverted, preceding the emergence of clinical signs; moreover, TP, WBC, eosinophil and platelet cell counts increased while monocyte and basophil cell counts decreased. After treatment, casualties and fistulas disappeared and both packed cell volume (PCV) and haemoglobin concentration significantly increased. Hp, SAA and ASG values were under the limit of detection or showed no significant differences.

**Conclusions:**

A role for captivity in contagion rate is suggested by the increase in antibody levels against *C. pseudotuberculosis* and the emergence of clinical signs. Although boosted by captivity, this is the first report of an outbreak of caseous lymphadenitis displaying high morbidity and mortality in wild ungulates. Treatment consisting of both vaccination and antibiotic therapy seemed to prevent mortality and alleviate disease severity, but was not reflected in the humoural response. Haematology and APP were not useful indicators in our study, perhaps due to the sampling frequency. Presumably endemic and irrelevant in the wild, this common disease of domestic small ruminants is complicating conservation efforts for the Iberian ibex in north-eastern Spain.

## Background

*Corynebacterium pseudotuberculosis* causes widespread abscesses in the lymph nodes, subcutaneous tissues and internal organs of buffaloes, wild ruminants, camels, cattle and horses [1-3]. It is the causative agent of caseous lymphadenitis (CLA), a disease occurring worldwide that primarily affects domestic small ruminants [4]. In wildlife, *C. pseudotuberculosis* has been previously described as isolated cases in pronghorns (*Antilocapra americana*) [5], elk (*Cervus elaphus canadensis*) [6] and Arabian oryx (*Oryx leucoryx*) [7], or as chance findings during routine meat inspection of antelope carcasses from a South African game reserve [8]. Human infection is also possible but unusual, being mostly related to occupational exposure [9]. *C. pseudotuberculosis* infection occurs through the oral, nasal and ocular mucosa, or through skin wounds. The bacterium can survive up to eight months in faeces, fomites or in the soil, a fact that favours its maintenance in small ruminant flocks worldwide. The principal clinical sign of CLA in sheep and goat is lymph node abscessation, which may be closed or fistulised, discharging pus and contaminating the environment. Infected animals without clinical signs also can shed bacteria through their respiratory tract and mechanical vectors such as flies have also been described to play a role [1,10].

Economic losses due to CLA are an important issue in the sheep and goat industries because of the reduction in wool, meat and milk production, the decrease of reproductive rates and the losses due to condemnation of carcasses and skins in abattoirs [1,10]. Lamentably, classical serological tests show inadequate sensitivity or specificity to detect contact with *C. pseudotuberculosis* [1], making disease detection difficult. Nevertheless, some enzyme-linked immunoabsorbent assay (ELISA) diagnostic tests have been observed to be effective in control and eradication programs, especially those based on phospholipase D (PLD) as an antigen [11,12]. Recently developed ELISA tests based on the detection of gamma interferon (IFN-γ) appear to be more sensitive in detecting early infection [12,13].

Disease management in sheep flocks is based on lancing and flushing-out or the surgical removal of lesions accompanied by topical disinfection and parenteral antibiotic treatment for 4–6 weeks [4,10]. Special care must be taken to prevent environmental contamination during drainage of the abscesses [10]. The effectiveness of such management measures strongly relies on the close surveillance of new infections, which is very difficult due the above-mentioned limitations in disease diagnosis. Vaccination has been the main strategy for CLA control in countries where the disease is endemic. Several vaccines have been developed and used with varied outcomes. The earliest ones consisted of bacterin-based vaccines, which initially displayed limited efficacy [14] but have improved over time [15,16]. The ability of PLD to protect against CLA encouraged toxoid vaccine studies [17], and combined bacterin-toxoid vaccines have also been used with success [18]. Live vaccines also elicit strong humoural and cell-mediated immune responses in goats and sheep [1,19]. Finally, the use of autogenous vaccines has been supported both in the literature and by veterinary practitioners for the treatment of CLA at herd level [20].

The Iberian ibex (*Capra pyrenaica*) is an endemic wild ruminant of the Iberian Peninsula, whose range is expanding both in Spain and Portugal [21-23]. The Red List of Threatened Species of the International Union for Conservation of Nature (IUCN) considers the status of *C. pyrenaica hispanica* to be of Least Concern (LC) [23]. However, threats such as fragmentation, competition with domestic livestock and other introduced wild ungulates or illegal hunting occur [24]. In addition, diseases have been one of the main factors responsible for demographic changes in the recent history of this species. The impact of sarcoptic mange, caused by *Sarcoptes scabei,* has been of particular relevance, causing mortalities of over 95% in ibex populations in southern Spain [25]. Other pathogens, such as *Mycoplasma agalactiae*, have caused less severe outbreaks in some Iberian ibex populations [26]. This is both a conservation issue and an economic concern in those regions where game exploitation (trophy hunting) of this valuable species occurs [27]. Hence, because of the biological value of this species and the ever present risk of game cessation due to disease outbreaks, the creation of stock reservoirs of Iberian ibexes have been promoted by the environmental agencies of the Spanish government in the main areas of distribution of this species (i.e., Sierra Nevada, Sierra de Cazorla and Serranía de Cuenca Natural Parks in southern and central Spain, respectively). In 2010, the threatening south–north advance of sarcoptic mange in the Iberian Peninsula prompted the decision to create a new stock reservoir of Iberian ibexes in the, up to date, sarcoptic mange-free subpopulation of the National Game Reserve of the Ports de Tortosa i Beseit (NGRPTB). This study analyzes the role of captivity in the development of a CLA outbreak that occurred in this new captive population and evaluates the effect of a CLA vaccine-based treatment on the occurrence of clinical signs, the immune humoural response, changes in haematological parameters, and acute phase protein (APP) concentrations.

## Materials and methods

### Study area and period

The study area is situated in the NGRPTB (40°48’28”N, 0°19’17”E), in Catalonia, north-eastern Spain. Specifically, this study was carried out in an enclosure of 17 hectares within the NGRPTB aimed at housing a stock reservoir of Iberian ibex. The facilities consist of a double peripheral fence, to prevent contact with animals outside of the fence, a quarantine area, a warehouse and a smaller fenced area with troughs and connected to a capture corral trap. The study was carried out between March 2010 and January 2013.

### Animals and samples

#### The founding ibex population

Eighteen ibexes were captured in 2010 (t0) by means of seven fixed box-traps scattered throughout the NGRPTB. Once trapped, the ibexes were darted by means of a blowpipe and anaesthetized with a combination of xylazine (3 mg/kg; Xilagesic® 20%, 200 mg/ml, Laboratorios Calier, Barcelona, Spain) and ketamine (3 mg/kg; Imalgene® 1000, 100 mg/ml, Merial, Lyon, France) [28]. One ibex died during transport from the box-trap site to the enclosure. Another ibex died once in captivity (between t0 and t1) and two escaped from the enclosure before the first handling (t1). On the other hand, two newborn ibexes from females captured pregnant in the wild added to the founding population within the enclosure between t0 and t1. Hence, sixteen ibexes, ten females and six males aged one to eleven years, were used for our longitudinal studies. Two of these ibexes were euthanized between t1 and t2 due to draining fistulas and in order to prevent disease spread. See Table 1 to ease understanding. All the death and euthanized ibexes were necropsied. Age of adult ibexes captured in the wild was determined by horn segment counts [29]. Individual identification was done by means of both ear tags and subcutaneous passive injectable transponders of 32 × 3.8-mm placed in the axillary area (Model Ri-Trp-RC2B, Tiris, Texas Instruments, Almelo, The Netherlands).

**Table 1:** **Result of the clinical examination performed in every ibex (ID) at each handling time (t0-t5) expressed as lesion status: FL: Presence of fistulas; GLS: generalized lymph node swelling; LLS: Local lymph node swelling; NL: No apparent affectation; ND: No data**

**ID**	**t0**	**t1**	**t2**	**t3**	**t4**	**t5**
**1**	NL	Died during transport				
**2**	NL	Died into enclosure				
**3**	NL	Escaped from enclosure				
**4**	NL					
**5**	NL	NL	Euthanatized into the enclosure			
**6**	NL	FL				
**7**	NL	FL	NL	NL	NL	NL
**8**	NL	GLS	NL	NL	NL	NL
**9**	NL	GLS	NL	NL	NL	NL
**10**	NL	GLS	GLS	LLS	NL	NL
**11**	NL	LLS	LLS	LLS	NL	NL
**12**	NL	LLS	LLS	NL	NL	NL
**13**	NL	LLS	GLS	LLS	NL	NL
**14**	NL	ND	FL	GLS	NL	NL
**15**	NL	ND	LLS	LLS	NL	NL
**16**	NL	LLS	LLS	LLS	NL	NL
**17**	NL	ND	FL	GLS	LLS	ND
**18**	NL	GLS	ND	LLS	NL	NL
**19**	New born	NL	LLS	LLS	LLS	GLS
**20**		LLS	LLS	LLS	NL	ND

#### Management of the CLA-infected ibex stock reservoir

**Figure 1:**
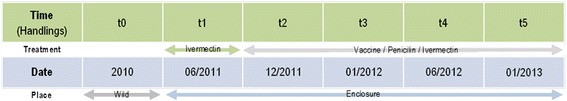
**Diagram of the handlings dates with the corresponding treatment and location of the ibexes at each handling time.**

Routine handlings began in June 2011 (t1) and were initially planned once a year. Nevertheless, owing to the CLA outbreak and the commencement of treatment, the second and third handlings took place in December 2011 (t2) and January 2012 (t3), respectively, and continued at six month intervals until January 2013 (t5). See the detailed chronology in Figure 1. To carry out handlings, the ibexes were captured, physically restrained, blindfolded, and held in 4x4 cm mesh sack nets (Ziboni Ornitecnica®, Bergamo, Italy). The ibexes were then concentrated in a handling zone and treated with acepromazine maleate (0.1 mg/kg; Calmo Neosan®, 5 mg/ml,, SmithKline Beecham, Madrid, Spain) to alleviate capture stress [30]. The handling protocol included collection of blood, administration of ivermectin (0.2 mg/kg sc; Ivomec® 1%; Merial, Toulouse, France), checking of ear tags and microchips and an external clinical examination. After CLA emergence, ibexes were also vaccinated and treated with penicillin G benzatine (1.2 million IU, intramuscular; Benzatard®; Syva, León, Spain) in each handling. Blood samples were collected from the jugular vein with disposable 10 ml syringes fitted with 0.8 × 25 mm. needles and processed within 12 h of collection. Two millilitres of each sample were placed in commercial tubes with tripotassium ethylenediaminetetraacetic acid (EDTA K_3_) as an anticoagulant. The remaining volume was placed in tubes for serum collection, allowed to clot at room temperature and centrifuged at 1800 *g*. Serum was frozen at −20°C until testing. The purpose of clinical examination was mainly to evaluate lymph node swelling (size and texture) and included palpation and visual inspection of the superficial lymph nodes of the head, neck, shoulders and hind limbs. To reduce variability and subjectivity, all examinations were done by the same person (GM, veterinary). Four clinical categories were established in order to perform the lesion score: ibexes with no apparent lesions (NL), local lymph node swelling (LLS), generalized lymph node swelling (GLS) and ibexes with the presence of fistulised lymph nodes (FL).

### Laboratory analyses

#### Bacteriology

Selected samples were collected from the four necropsied ibexes and from a fifth live ibex that healed after swabbing when presenting with a fistula during the t2 handling. Samples were directly cultured on blood agar base supplemented with 5% sterile defibrinated sheep blood (Oxoid S.A., Spain). Colonies obtained after 24–48 h of aerobic incubation at 37°C were Gram-stained. Colonies of bacteria with morphological characteristics compatible with *C. pseudotuberculosis* were tested using API Coryne Strip (API CS; bioMérieux, France) according to the manufacturer’s recommendations. The API CS method involves a battery of 20 tests and the results are interpreted using the API web system (see: https://apiweb.biomerieux.com/servlet.)

#### Serologic analyses

An indirect ELISA to detect *C. pseudotuberculosis* antibodies was performed according to Solanet *et al.* [31]. Two different stumps were used to acquire the antigens; one from CLA-infected farm goats from the Region of Murcia, south-eastern Spain, and the other from the CLA-infected ibexes. Six sera from domestic goats of farms free of paratuberculosis, tuberculosis and pseudotuberculosis in the Region of Murcia (Spain) were used as negative controls. Five serum samples from CLA clinical cases confirmed by histopathological and bacteriological analyses in domestic goats from the Region of Murcia were used as positive controls. Optical density (OD) was corrected by blank well lecture, and the cut-off values were calculated as the mean of negative sera OD (blank corrected) + 3 S.D. Another ELISA was done to detect antibodies against *Mycobacterium bovis* [32] using as the antigen the recombinant protein MPB70 from *Mycobacterium bovis* provided by Jim McNair (Belfast, Northern Ireland, United Kingdom). A cut-off value was calculated using ten sera obtained from ten goats in a tuberculosis-free herd (Experimental Farm, School of the Veterinary Medicine, Murcia). These negative control animals previously tested negative for tuberculosis by skin test and the IFN-γ release assay.

Finally, a commercial ELISA kit was used to detect antibodies against *Mycobacterium avium* subsp. *paratuberculosis* -MAP- (Parachek®, Prionics AG, Zurich, Switzerland). Results were expressed as OD after reading the plates at 450 (*C. pseudotuberculosis*) or 405 (*M. bovis* and MAP) nm in an ELISA plate reader (DigiScan with DigiWin Software, ASYS Hitech, Austria).

#### Haematology

The erythrocyte (RBC), leukocyte (WBC), platelet (PLT) and differential WBC (lymphocytes [Lymph], neutrophil [Neut], eosinophil [Eos], monocyte [Mono] and basophil [Baso]) counts, and the haemoglobin concentration (Hb) were determined by means of an automated laser analyzer (Advia 120®, Bayer, Fernwald, Germany). The packed cell volume (PCV) was measured using a microPCV centrifuge (Hematospin 1400, Hawksley, Lancing, England) at 14000 *g* for 6 min. The total protein concentration (TP) was determined by refractometry (Euromex RD 712 clinical refractometer; Euromex, Arnhem, Netherlands).

#### Acute phase protein

Serum concentration of haptoglobin (Hp) was quantified using a commercial spectrophotometric assay (Phase Haptoglobin assay, Tridelta Development Ltd, Ireland). Serum amyloid A (SAA) concentration was determined with a commercial solid phase sandwich ELISA (Phase SAA assay, Tridelta Development Ltd, Ireland). Analyses were performed according to the manufacturer’s instructions and the final absorbance measured in a microtitre plate reader (PowerWave XS, Bio-Tek Instruments Inc., Vermont, USA) at 630 nm and 450 nm for Hp and SAA, respectively. These analytical determinations were recently validated [33].

Acid soluble glycoprotein (ASG) concentration was also quantified as it correlates well with alpha-1-acid glycoprotein (AGP) in most species [34]. The ASG component of the serum samples was determined using the method described in Winzler (1955) [35], modified by Nagahata *et al*. [36] and Eckersall *et al.* [34], and optimized by Tecles *et al.* [37]. Briefly, serum was precipitated by mixing with perchloric acid and then centrifuged at 1750 *g* for 10 min. Next, 20 μl of supernatant was mixed with 196 μl of the reagent bicinchoninic acid (Sigma chemical Company, St. Louis, Missouri, USA), the mixture incubated at 37°C for 25 s, and then 4 μl of copper sulphate (Sigma) added as a start reagent. The protein content in the supernatant was then measured after 325 s with a Cobas Mira Plus analyzer (ABX, Montpellier, France) at a wavelength of 550 nm. Commercial purified bovine α1-acid glycoprotein (AGP; Sigma) was used as a standard. A stock solution was prepared by diluting 25 mg of bovine AGP in 5 ml of distilled water and a standard curve performed by mixing the stock solution with perchloric acid at a 1:4 dilution.

### Therapeutic intervention

An autovaccine containing 1 × 10^8^ cfu/ml of the *C. pseudotuberculosis* isolated from the CLA-infected ibexes, and a strain of *Archanobacterium haemolyticum* previously isolated at the Department of Animal Health at the University of Córdoba, which presents strong immunogenic properties against other corynebacterial species, was elaborated (unpublished data). Briefly, *C. pseudotuberculosis* and *A. haemolyticum* isolates were cultivated in brain heart infusion (Oxoid, Spain) at 37°C for 24 h and inactivated with 0.3% formaldehyde (30-40% w/v, Panreac, Spain). The suspension was centrifuged at 720 *g* for 15 min at 4°C and the pellets re-suspended in an isotonic saline solution until the pattern 0.5 in the McFarland scale was obtained. The vaccination scheme consisted of 5 ml subcutaneous (sc) doses (2 ml for juveniles), with the first dose on December 2011 (t2) followed by revaccination one month later (t3) and subsequent doses every 6 m, on July 2012 and January 2013 (t4 and t5, respectively). Penicillin G benzatine (1.2 million IU, intramuscular; Benzatard®; Syva, León, Spain) was administered to reduce infection progression. Penicillin G has demonstrated high action against *Corynebacterium* spp. in antibiograms (Personal communication).

### Statistical analysis

Changes in the physiological responses (i.e., changes in haematologic parameters and APP concentration) and in anti-*C. pseudotuberculosis* antibody levels (i.e., Optical Density obtained by means of ELISA) of ibexes were assessed by means of a multivariate analysis of variance (MANOVA). In our case, the physiological response was defined as a canonical derived response variable named haematologic parameters (RBC, WBC, PLT, WBC, Lymph, Neut, Eos, Mono, Baso, Hb), APP (Hp, SAA, AGP), and anti-*C. pseudotuberculosis* antibodies (O.D. CLA-ELISA) and the explanatory factors were the handling times (from t0 to t5). We performed three different analyses: the first aimed to assess the early captivity effect by comparing the anti-*C. pseudotuberculosis* antibody levels, and the haematology and APP values between t0 and t1; the second assessed the long-term captivity effect by comparing t0 with t1-t5; finally, for the assessment of the treatment effect, t2 (when the treatment started) was compared with t3 (one month later). Prior to any analysis, MANOVA assumptions (i.e., correlation between dependent variables, multivariate normality and absence of residual patterns), [38] were checked. All statistical analyses were carried out in the statistical software R version 3. 0. 3 (R Development Core Team 2014).

## Results

### Postmortem findings

The ibex that died during transport, the one that died in the enclosure and the two euthanized ibexes were necropsied and diagnosed with CLA on the basis of multiple abscesses and a positive *C. pseudotuberculosis* culture. Two additional free-living wild ibexes were diagnosed with CLA in 2005 and 2007 in the NGRPTB. All affected animals (four males, 3 to 15 years old and two females, 7 and 10 years old) had low body weights and widespread serous fat atrophy. The most frequently affected peripheral lymph nodes were those of the head (submandibular, 2/6) and neck (prescapular, 3/6). Amongst these six cases, four presented with internal dissemination of the infection with lesions seen in mediastinal lymph nodes and lungs (4/6) as well as in mesenteric lymph nodes (3/4), kidney (2/4), spleen (1/4) and liver (1/4).

### Analysis of lesions score

Results are presented in Tables 1 and 2. Clinical signs of CLA were not detected at t0, with pathological changes appearing between t1 and t5. Nine ibexes displayed either localized (n = 5) or generalized (n = 4) lymph node swelling at t1. Disease signs decreased after treatment, when no more fistulas were observed and only three ibexes displayed generalized infection. Finally, whereas generalized infection and fistulas were mostly associated with a positive CLA serological status, local and no apparent lesions were indifferently associated with a CLA positive or negative status. However, 22 out of the 24 (92%) clinical examinations where a NL status was associated with a positive CLA serological result belonged to individuals displaying clinical signs in previous examinations. On the other hand, at least three out of the 13 clinical examinations (23%) where a LLS status was associated with a negative CLA serological result belonged to ibexes seroconverting and/or displaying a GLS status later.

**Table 2:** **Lesions score: Number of clinical examinations performed in every handling divided by four lesion status: FL: Presence of fistulas; GLS: generalized lymph node swelling; LLS: Local lymph node swelling; NL: No apparent affectation; ND: No data**

		**Ibexes**	**FL**	**GLS**	**LLS**	**NL**	**ND**
t0		18	0	0	0	18	0
t1		16	2	4	5	2	3
t2		14	2	2	6	3	1
t3		14	0	2	8	4	0
t4		14	0	0	2	12	0
t5		14	0	1	0	11	2
Period	Wild (t0)	18	0	0	0	18(100%)	
	Captivity (t1-t5)	16	4(6.5%)	9(14%)	21(34%)	32(52%)	
Treatment	Before (t1-t2)	16	4(18%)	6(27%)	11(50%)	5(23%)	
	After (t3-t5)	14	0	3(7.5%)	10(25%)	27(67.5%)	
*CLA-ELISA*	Negative	20	0	2(22.2%)	13(61.9%)	26(52%)	
	Positive		4(100%)	7(77.8%)	8(38.1%)	24(48%)	

These clinical examinations are put into groups to show the number of ibexes observed with every lesion status and the percentage they represent (in brackets) before (t0) and once in captivity (t1, t2, t3, t4, t5), before (t1 and t2) and after introducing treatment (t3, t4, t5) and in relation to the CLA-ELISA results. The number of ibexes present in every handling or included in every group is shown (Ibexes).

### Laboratory results

**Figure 2:**
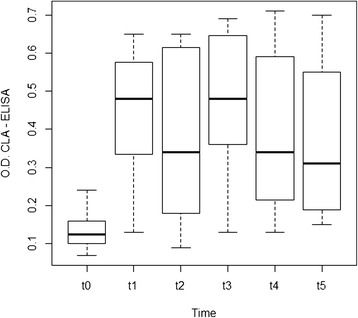
Box plot showing significant differences (p = 0.001**) between the mean optical density of the ibex CLA-ELISA processed samples at the moment of first capture in the wild (t0) and in the subsequent handlings (t1, t2, t3, t4, t5).

At least 12 out of 14 ibexes (85.7%) changed their serological status once within the enclosure, as shown by the significantly higher mean optical density in the CLA-ELISA from t1 to t5 (Table 3). The antibody levels did not show significant differences once in captivity (t1-t5) (Figure 2). Three ibexes did not seroconvert during the study period whereas one ibex displayed a positive ELISA result in t0. Two of the ibexes that seroconverted within the enclosure changed their serological status again to negative at some time between t2 and t4. In the *M. bovis* ELISA, only one ibex displayed a temporary positive result in t3 and, in the MAP ELISA, none of the ibexes displayed antibodies against *M. a.* subsp. *paratuberculosis*.

**Table 3:** **MANOVA and specific ANOVA showing influence of Iberian ibex captivity (t0 ibexes were in the wild, t1-t5 in the enclosure), and treatment (t2 and t3, before and after treatment, respectively) on physiological parameters (i.e., haematological parameters and acute phase protein concentration) and anti-** ***C.pseudotuberculosis*** **antibody presence (i.e., Optical Density (O.D.) obtained by means of ELISA) as a canonical response**

**MANOVA**	**Pillai’s trace**	***F-value***	***P-value***
Early captivity (t0 vs t1)	0.974	15.793	0.001
Long-term captivity (t0 vs t1-t5)	0.818	10.284	3.468e-08
Treatment (t2 vs t3)	0.735	1.979	0.141
ANOVA	Mean ± SE (Min-Max)	*F-value*	*P-value*
Early captivity			
*IgG* (O.D.)		37.993	6.352e-06
t0	0.22 ± 0.02 (0.16-0.4)		
t1	0.5 ± 0.04 (0.22-0.7)		
*Leukocytes* (×10^3^ cells/μl)		5.338	0.032
t0	11.2 ± 1.33 (5.56-21.98)		
t1	15.16 ± 0.86 (12.11-19.95)		
*Platelets* (×10^3^ cells/μl)		6.652	0.018
t0	260.82 ± 52.41 (46–563)		
t1	470.55 ± 58.3 (109–784)		
*Monocytes* (×10^3^ cells/μl)		7.370	0.014
t0	2.16 ± 0.58 (0.6-6.2)		
t1	0.59 ± 0.15 (0.2-2)		
*Eosinphils* (×10^3^ cells/μl)		5.085	0.036
t0	3.13 ± 0.67 (1–8.4)		
t1	5.08 ± 0.72 (1.8-10)		
*Basophils* (×10^3^ cells/μl)		10.604	0.004
t0	1.76 ± 0.37 (0.6-5.2)		
t1	0.59 ± 0.05 (0.4-1)		
*Total Protein* (g/dl)		15.952	0.001
t0	7.1 ± 0.13 (6.4-7.9)		
t1	8.31 ± 0.27 (7–9.6)		
*Long-term captivity*			
*IgG* (O.D.)		10.432	0.002
t0	0.23 ± 0.03 (0.17-0.4)		
t1-t5	0.48 ± 0.03 (0.196-1.37)		
*Basophils* (×10^3^ cells/μl)		56.797	1.644e-09
t0	1.38 ± 0.13 (0.6-1.7)		
t1-t5	0.61 ± 0.04 (0.2-1.2)		
*Treatment*			
*Haemoglobin* (g/dl)		4.689	0.041
t2	10.46 ± 0.46 (7.7-13.2)		
t3	11.92 ± 0.49 (8.6-13.6)		
*PCV* (%)		5.061	0.034
t2	33.25 ± 1.54 (24.5-42.7)		
t3	38.33 ± 1.65 (27.4-44.8)		

ANOVA values illustrate statistically significant parameters associated with captivity and treatment effect.

**Figure 3:**
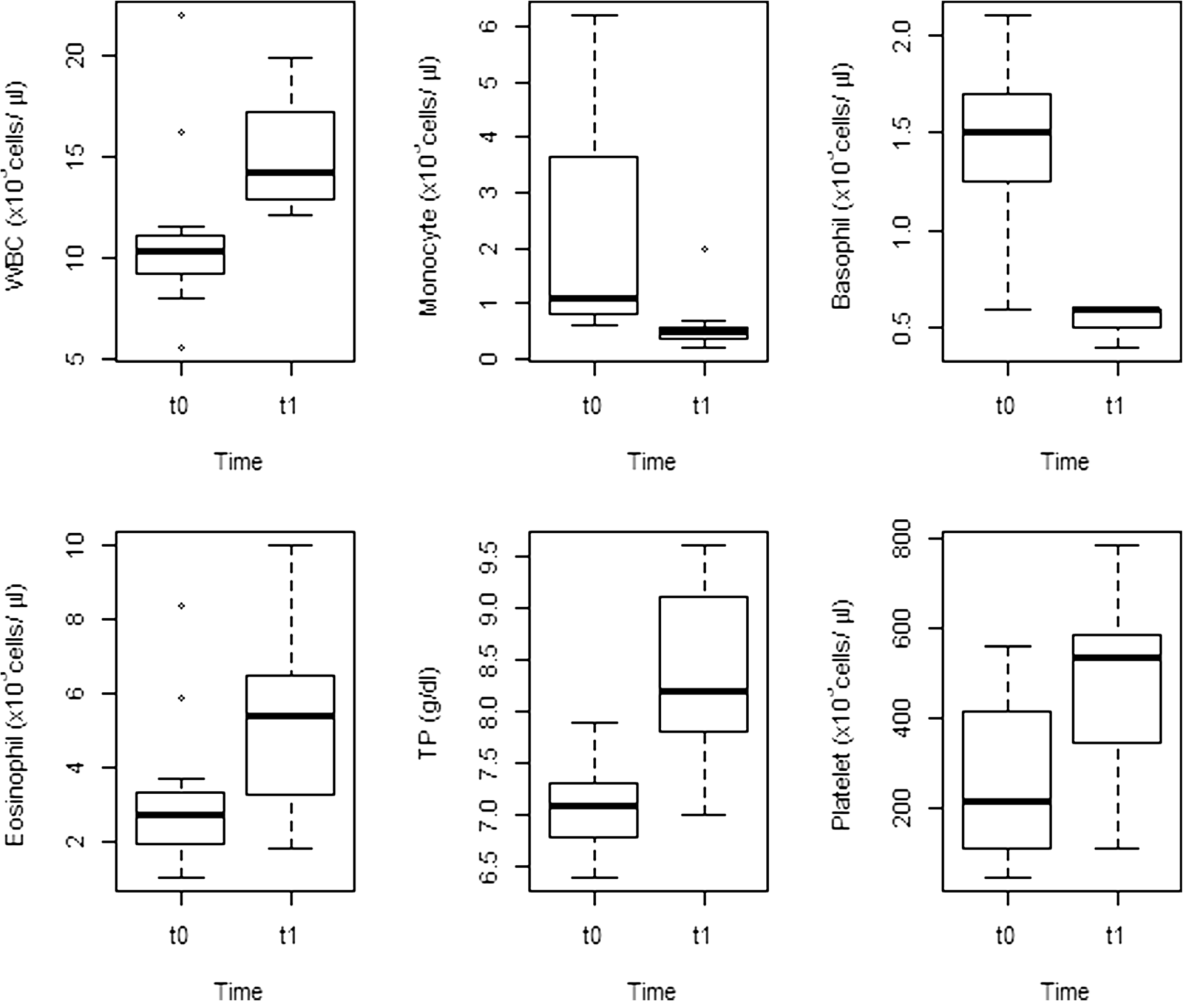
**Box plots showing significant differences (p < 0.05) in the mean leukocyte (WBC), monocyte, basophil, eosinophil and platelet counts, and in the serum total protein (TP) concentration of the ibexes at the moment of capture in the wild (t0) and at the first handling within the enclosure (t1).**

**Figure 4:**
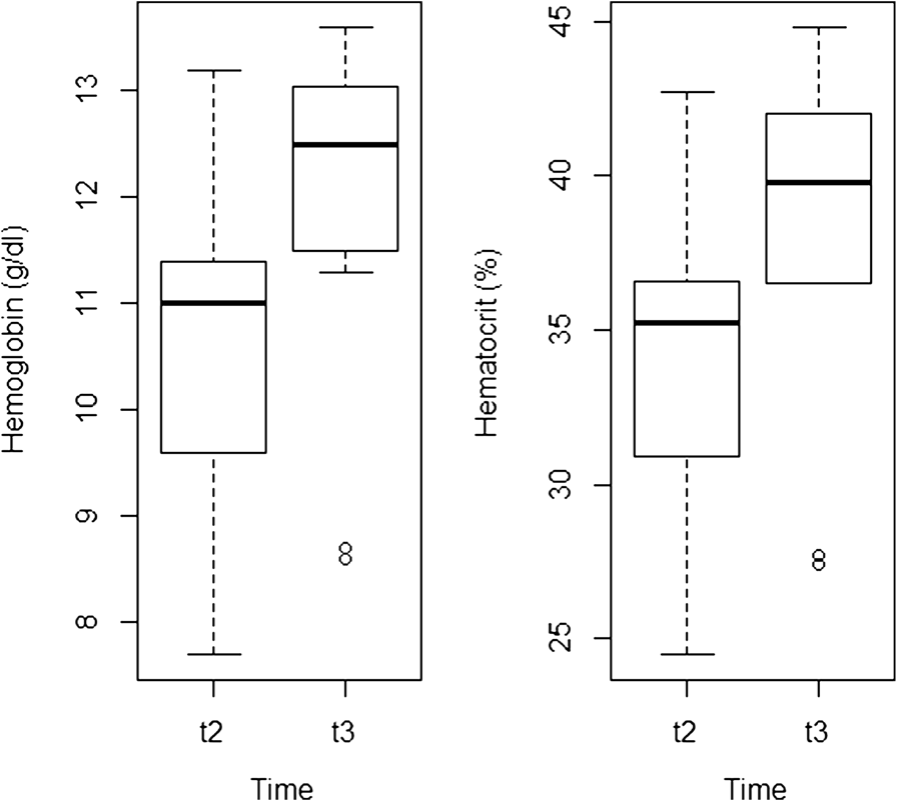
**Box plots showing the significant differences (p < 0.05) in the mean haemoglobin concentration and in the PCV of the ibexes just before the start of treatment (t2) and one month later (t3).**

TP, WBC, eosinophil and platelet cell counts increased (between one- and two-fold) whereas monocyte and basophil cell counts decreased (three-fold) from t0 to t1 (Figure 3). Both PCV and haemoglobin concentrations significantly increased (one-fold) from t2 to t3 (Figure 4). Table 3 illustrates the influence of both captivity and treatment in the physiological responses of ibexes.

All SAA values and most Hp values were under the limit of detection considered acceptable for interpretation. Only three samples displayed interpretable Hp values, which showed no significant correlation with any other variable. ASG values showed no significant differences according to time or lesions score or correlation with other parameters.

## Discussion

This is the first outbreak of CLA reported in Iberian ibex and, to our knowledge, in any other wild ungulate population. However, the infection rate leading to this outbreak of disease was favoured by captivity, as evidenced by the increase of the antibody levels against *C. pseudotuberculosis*, casualties and casuistry of lesions. Stress-induced immunosuppression caused by captivity may have favoured disease severity. The treatment including both antibiotics and vaccination apparently prevented casualties and fistulas but was not reflected in the humoural immune response. The ibex displaying a positive status for antibodies against *C. pseudotuberculosis* at t0, when first captured in the wild, may have acted as a source of infection within the enclosure. In fact, this ibex was found dead seven months after being placed in captivity and CLA was diagnosed as the cause of death. Other ibexes with subclinical affectation could have gone unnoticed in the clinical exploration performed at t0. Once within the enclosure, ibexes developing visible clinical signs such as draining fistulas could have gone unnoticed for weeks between handlings, contaminating the environment due to the lack of daily on-site staff presence. Mutual grooming, head butting, inquisitive attitudes or the habit to scratch shoulders against hard surfaces, are typical behavioural characters of goats that increase the risk of contagion [4,39], and other factors may have played a role in the evolution of this CLA outbreak as well. *C. pseudotuberculosis* displays high levels of survival in the environment [3] and the mechanical transmission of this bacterium by houseflies and other Diptera has been described in cattle and buffaloes [1,10].

Lesion score is the only evidence of a clear health improvement after treatment administration in the ibexes of our study. This is also observed in other vaccine studies [40]. ELISA classified as positive some serum samples from ibexes without clinical lesions; most of these ibexes had suffered from the disease previously and had recovered. The two individuals where this condition was not confirmed could either have gone unnoticed between handlings, suffered a subclinical affectation or could have been false positive results. Hence, whenever available, the medical record is important to interpret the ELISA results. In addition, the clinical external examination performed in the studied ibex population, based on the assessment of normality of lymph node size and swelling, may not be sensitive enough to detect animals with low grade lesions. The necropsied ibexes had abscesses, most frequently, in the lymph nodes of the anterior body part, which agrees with previous reports [4,10,39,41]. However, these studies did not observe a high percentage of visceral lesions (66.7% in our study). Thus, improved methods for clinical assessment are needed. On the other hand, the ELISA also classified as negative an important percentage of clinically affected ibexes. Since CLA is a chronic condition, the ibexes in the initial phase of infection may not have had time to develop a detectable antibody response. This may have occurred in at least the three ibexes displaying more severe affectations and seroconverting later. However, false negative results derived from inadequate sensitivity cannot be discarded. The ELISA used was described as highly sensitive (98%) and specific (100%) in CLA-affected lambs [31]. These values were lower in our study. However, despite unknown sensitivity and specificity, ELISA is considered a suitable option for the detection of *C. pseudotuberculosis* infection in Iberian ibexes.

The lack of correlation between treatment and the humoural response contrasts with the increase in antibody levels observed in domestic small ruminants immunized with bacterin vaccines [42], toxoid-based vaccines [43] or combined vaccines [18]. Immunity against *C. pseudotuberculosis* is described as complex, involving both cellular and humoural immune responses. In the last several years, IFN-γ and cytotoxic T-cells have been described as the main factors responsible for the protection against *C. pseudotuberculosis* [40,44]. Therefore, the absence of an antibody response to vaccination may not be interpreted as a lack of vaccine effect. Aqueous adjuvants have been shown to diminish vaccine efficacy [45]. We cannot separate the effects of vaccines and those of antibiotics, as all the ibexes received both treatments and there was no control group. This was unavoidable as this was not a designed experiment, but a management intervention. However, the treated ibexes displayed significant health improvement at handlings t4 and t5, preceded by six months without any treatment. An antibiotic-only treatment is not expected to have such a long lasting effect, which indicates a vaccine effect. The lack of seroconversion in three ibexes suggests individual differences in immune response. However, a lack of sensitivity of the ELISA test used could also explain this. The possibility of cross-reactions with *M. bovis* and *M. a. paratuberculosis* [3,46] was discarded with specific ELISAs. Previous studies have not found tuberculosis in Iberian ibexes from our study area [47,48]. Thus, the *M. bovis* ELISA positive ibex in this study is probably a false positive as other samples of the same ibex collected later displayed a negative result.

Apart from higher basophil and eosinophil cell counts, the mean haematological parameters of the ibexes included in our study were similar to the reference values previously described [49,50]. Basophils apparently decreased once in captivity but the factors determining the observed decrease are unclear. In fact, controversy remains on the real factors determining basophil counts and this is a difficult-to-assess parameter in veterinary clinical pathology. Since it is a minority blood cell type, small differences in count may be observed as large variations. Hence, the observed differences could be due to differences in the capture and analytical methods. The increase of WBC and eosinophil cell counts could be related to both CLA (bacterial-induced leukocytosis) and higher parasite load derived from captivity conditions (parasite-induced eosinophilia). Parasite-induced eosinophilia is a well-known phenomenon [51,52]. Previous studies reported high prevalences and intensity of parasitosis by ixodides and helminthofauna in NGR Ports of Tortosa Beseit [53]. Coprology did not reflect a marked increase in parasitic load. Conversely, high tick loads were observed during handlings. Increased serum total protein concentration may reflect increased serum immunoglobulins (antibodies anti-*C. pseudotuberculosis*) and platelet counts may increase as a result of wounds caused by extremely high tick loads. Monocyte cell counts were also higher with regard to values previously published [49], which could be associated with stress induced by the box-trap capture method or to subclinical inflammatory conditions [54]. Although the leukogram is considered a useful tool for monitoring infectious processes, ruminants may show no or few changes even during severe infections [55]. Haemoglobin concentration and PCV increased from t2 to t3. This could be explained by an increased stress response due to proximity between t2 and t3 handlings, sensitization and increased catecholamine release in t3 [56]. However, it could also be due to the start of treatment in t2 and improved health of ibexes in t3.

The acute phase reaction is a physiological response to infection, inflammation, injuries or stress and includes the increase or decrease in the expression of a protein family called APP. Hence, the quantification of APP can be a way to determine the health status of an animal or a herd [57] and thus be used to assess disease pathogenesis, the evolution of an infection or the efficacy of drugs and vaccines. For example, CLA-affected sheep have been described to display higher serum concentrations of Hp, SAA and AGP [55,58]. Although the validation of analytical methods and reference values of SAA and Hp in Iberian ibex have been provided recently [33], we were not able to detect significant changes in Hp, SAA nor ASG. Hp and SAA have been proposed as major acute phase proteins in CLA-affected sheep and ASG is considered a moderate acute phase protein in chronic stages [58]. Although interspecific differences may exist, the frequency of sampling was lower in our study and changes in the APP concentration may have gone unnoticed due to the spaced sampling.

## Conclusion

This is the first outbreak of CLA in Iberian ibex described in the literature, displaying high morbidity and mortality. However, captivity conditions had an important role over the course of the disease. This makes clear the need for improved methods for early detection of infected animals and quarantine protocols. The CLA-ELISA test used in the present study can be a useful tool for decision-making during quarantine management. However, sensitivity and specificity should be further assessed.

Treatment consisting of both vaccination and antibiotic therapy seemed to prevent mortality and alleviate disease severity, although this was not reflected by the humoural response. An experimental design aimed at differentiating the effect of vaccine from that of antibiotics was not possible in the present study. Despite the fact that time lapses between consecutive handlings (and treatments) point to a vaccine effect, an experimental design would be desirable if future studies are launched to assess vaccine efficacy.
